# Hyalinizing trabecular tumor of the thyroid gland

**DOI:** 10.4103/0970-9371.70741

**Published:** 2010-04

**Authors:** Sumiti Gupta, Shilpi Modi, Veena Gupta, Nisha Marwah

**Affiliations:** Department of Pathology, Pt. B.D. Sharma PGIMS, Rohtak, India

**Keywords:** Fine needle aspiration cytology, hyalinizing trabecular tumor, thyroid

## Abstract

Hyalinizing trabecular tumor (HTT) is an unusual and controversial lesion of the thyroid gland. Some have considered it a unique entity, some have considered it a variant of papillary carcinoma, and still others have considered it a nonspecific pattern that may be seen with a variety of thyroid lesions. The histological and ultrastructural characteristics of this thyroid neoplasm are well documented; however, its cytological diagnosis by fine needle aspiration cytology (FNAC) remains challenging. The cytomorphological features of this entity overlap with both papillary and medullary carcinoma to a varying extent. We report a case of HTT with cytological evaluation by FNAC in a 28-year-old male.

## Introduction

Hyalinizing trabecular tumor (HTT) of the thyroid gland is a rare neoplasm first described by Carney *et al*.[1] in 1987 as hyalinizing trabecular adenoma. After the original proposal by some authors,[2] the term adenoma was replaced by “tumor” in the recent WHO classification of tumors of endocrine organs, which defined this lesion as “a rare tumor of follicular cell origin with a trabecular pattern of growth and marked intratrabecular hyalinization”.[3] The cytological recognition of this unusual tumor is difficult as it shows some features of papillary thyroid carcinoma (PTC) and other features of medullary thyroid carcinoma (MTC). Here, we relate our experience in one such case.

## Case Report

**Figure 1:**
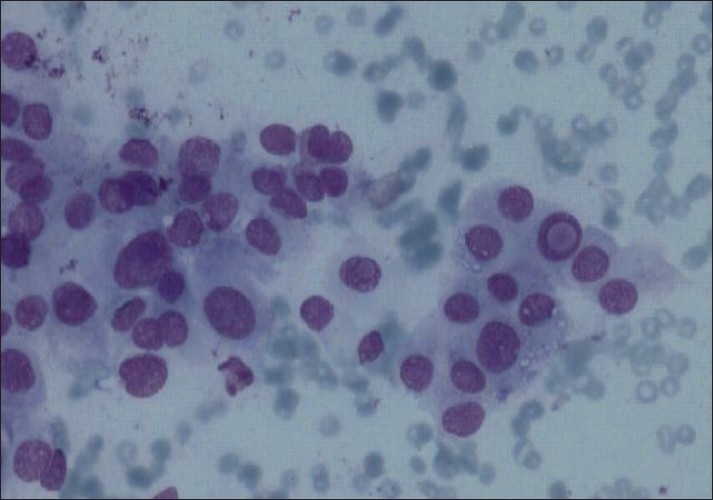
Fine needle aspiration cytology smear showing the cluster of tumor cells revealing nuclear overlapping and intranuclear inclusions (Giemsa, ×400)

**Figure 2:**
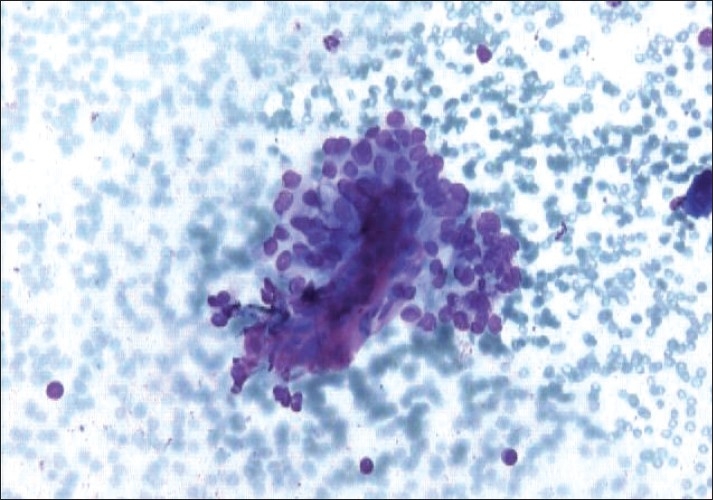
Fine needle aspiration cytology smear showing fragments of acellular hyaline material within the tumor cell cluster (Giemsa, ×400)

**Figure 3:**
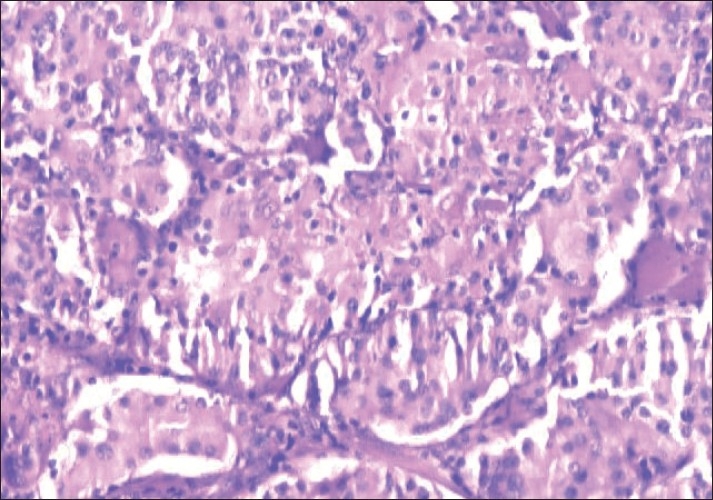
Histology of the tumour showing trabeculae and nests of tumor cells separated by hyalinized stroma (Haematoxylin and Eosin, ×200)

A 28-year-old male presented with swelling of left lobe of the thyroid, gradually increasing in size, for 1 year. On examination, the swelling was nodular, measured 3 cm×2 cm and was firm, nontender, and moving with deglutition. Serum T_3_, T_4_ and TSH were within normal limits. Ultrasonography showed a large hypoechoic lesion of 3.1 cm×1.1 cm in the left lobe of the thyroid along with calcification. Fine needle aspiration cytology (FNAC) was performed using a 23-gauge needle and disposable 10 ml plastic syringe. The smears prepared were air dried and stained with Giemsa stain. The smears were moderately cellular. The epithelial cells were present in small clusters and dispersed singly and showed abundant filamentous, vacuolated, and ill-defined cytoplasm. The nuclei were oval and slightly pleomorphic with occasional conspicuous nucleoli. Nuclear overlapping, nuclear grooves, and intranuclear cytoplasmic inclusions were noted in several groups [Figure 1]. Small and irregular fragments of acellular hyaline material were observed within a few cell clusters [Figure 2]. Congo red staining was negative. Diagnosis of neoplastic lesion with possibilities of HTT and papillary carcinoma were suggested. Left thyroid lobectomy was performed. Histological examination of the specimen revealed a well-circumscribed tumor composed of tumor cells arranged in trabeculae and small nests separated from each other by hyalinized stroma. The cells showed abundant hyalinized cytoplasm and slightly pleomorphic elongated nuclei. Nuclear grooves were present in most nuclei. Some of the nuclei also showed intranuclear inclusions [Figure 3]. Final diagnosis rendered was HTT. The patient is on regular follow-up and is doing well with no evidence of tumor recurrence or metastasis.

## Discussion

HTT is a unique neoplasm of follicular derivation that usually arises in females. The most recent and controversial debate surrounding HTT concerns its potentially malignant behavior and the possible relationship to PTC. Most experts consider this tumor to be benign. Based on this, all cases of HTT that show classic morphology, as described by Carney *et al*.,[1] fail to show unequivocal capsular and/or vascular invasion and have not metastasized. Some authors have recently proposed that these tumors actually represent a variant of PTC due to similar nuclear cytology, immunoprofile and RET oncogene rearrangements in both tumors. In addition, some authors have also suggested that this is not a distinct entity because a similar growth pattern can be seen in other primary and secondary tumors of the thyroid. Because of this debate, most experts designate these tumors as HTT.[2,4,5]

HTT occur as solitary or multiple nodules. They are well circumscribed and encapsulated. The classic trabecular pattern with hyalinization of the tumor cell cytoplasm and matrix and the presence of nuclei with prominent grooves and cytoplasmic inclusions are the main histological features of HTT.[6]

By immunohistochemistry, HTT is positive for thyroglobulin and TTF-1. Cytokeratin-19 and high molecular weight cytokeratin are usually negative in HTT whereas they show a variable pattern of galectin-3 expression.[4] Hirokawa and Carney[7] stated that unique cytoplasmic MIB-1 (Ki-67) expression in HTT is useful in making the distinction from PTC. Casey *et al*.[8] described the application of this finding to thyroid fine-needle aspirates.

HTT is misdiagnosed almost uniformly in FNAC specimens because of the confusing similarity of its nuclear features to those of PTC and the presence of a misleading hyaline material that mimics amyloid, often being misdiagnosed as MTC.

Some authors have proposed a cytological distinction between HTT and papillary carcinoma of the thyroid, but its cytological diagnosis remains challenging.[6,9,10]

Kuma *et al*.[6] observed that the radial arrangement of the tumor cells surrounding the hyaline material, vague, curved nuclear palisading, spindled or elongated cells, ill-defined cell border, faintly stained, filamentous cytoplasm, and hyaline material in the background are useful in diagnosing HTT and distinguishing it from papillary carcinoma. A lack of papillary architecture and sheet-like arrangement also suggests HTT.

Goellner and Carney[9] stated that spindle-shaped nuclei, perinucleolar halo, amorphous hyaline material, and whorling parallel arrays suggest HTT whereas papillary configuration suggests papillary carcinoma.

Casey *et al*.[10] stated that the combination of a bloody background, radially oriented cohesive cells, cells with abundant cytoplasm, nuclei with very frequent cytoplasmic inclusions and grooves, and the presence of hyaline should suggest the presence of HTT.

HTT can also pose diagnostic difficulties in the differentiation from MTC. The presence of elongated and spindled cell forms, dispersed cellularity, and hyaline acellular material in the aspirates of some of these lesions can lead to the diagnosis of medullary carcinoma. The presence of amorphous hyaline material in the aspirate may suggest amyloid. These tumors can be distinguished from MTC by Congo red negativity, positive thyroglobulin immunoreactivity, and negative calcitonin immunoreactivity.[9]

In our case, the nuclear features were the main reason for considering the diagnosis of papillary carcinoma in the cytological material. However, a tightly cohesive sheet-like arrangement of the tumor cells and papillary configuration, which are usually seen in papillary carcinoma, were not seen. Also psammoma bodies, viscous colloid, and multinucleate giant cells were absent. Compared with medullary carcinoma, the amorphous material in this case appeared less solid and well-defined than amyloid and did not contain the streaked nuclear material that is often associated with aspirated amyloid. Cytoplasmic granules, as described in medullary carcinoma, were not observed.

To conclude, cytological, it is difficult to differentiate HTT from papillary and medullary carcinoma. The cytological diagnosis of HTT should be considered in thyroid lesions with nuclear grooves and/or pseudoinclusions that lack other diagnostic criteria for papillary carcinoma.
